# TLR3-/4-Priming Differentially Promotes Ca^2+^ Signaling and Cytokine Expression and Ca^2+^-Dependently Augments Cytokine Release in hMSCs

**DOI:** 10.1038/srep23103

**Published:** 2016-03-16

**Authors:** Kyoung Sun Park, Sun Hwa Kim, Amitabh Das, Shao-Nian Yang, Kyoung Hwa Jung, Mi Kyung Kim, Per-Olof Berggren, YoungSeek Lee, Jin Choul Chai, Hyun Jin Kim, Young Gyu Chai

**Affiliations:** 1 Department of Molecular and Life Sciences, Hanyang University, Ansan, Korea; 2 Department of Physiology, Sungkyunkwan University School of Medicine, Suwon, Korea; 3 The Rolf Luft Research Center for Diabetes and Endocrinology, Karolinska Institutet, Karolinska University Hospital L1, SE-171 76 Stockholm, Sweden

## Abstract

In human mesenchymal stem cells (hMSCs), toll-like receptor 3 (TLR3) and TLR4 act as key players in the tissue repair process by recognizing their ligands and stimulating downstream processes including cytokine release. The mechanisms of TLR3- and TLR4-mediated cytokine releases from hMSCs remain uncertain. Here, we show that exposure to the TLR3 agonist polyinosinic-polycytidylic acid (poly(I:C)) or incubation with the TLR4 agonist lipopolysaccharide (LPS) increased the mRNA expression levels of TLR3, TLR4 and cytokines in hMSCs. Poly(I:C) exposure rather than LPS incubation not only elevated inositol 1,4,5-triphosphate receptor (IP_3_R) expression and IP_3_R-mediated Ca^2+^ release, but also promoted Orai and STIM expression as well as store-operated Ca^2+^ entry into hMSCs. In addition, we also observed that 21 Ca^2+^ signaling genes were significantly up-regulated in response to TLR3 priming of hMSCs by RNA sequencing analysis. Both poly(I:C) and LPS exposure enhanced cytokine release from hMSCs. The enhanced cytokine release vanished upon siRNA knockdown and chelation of intracellular Ca^2+^. These data demonstrate that TLR3- and TLR4-priming differentially enhance Ca^2+^ signaling and cytokine expression, and Ca^2+^ -dependently potentiates cytokine release in hMSCs.

Human mesenchymal stem cells (hMSCs) are not only capable of self renewal and differentiation into osteoblasts, chondrocytes, adipocytes, myocytes and even neurons^1^,^2^, but they are also capable of creating a local immunosuppressive milieu that is heavily dependent on toll-like receptors (TLRs)^3^,^4^,^5^,^6^. It is well known that TLRs are expressed in immunocytes such as macrophages and dendritic cells, where they function as crucial sentinels of the innate immune system by recognizing structurally conserved molecules derived from microbes^7^,^8^,^9^. Moreover, TLRs are also ubiquitously present in other human tissues including hMSCs^4^,^10^,^11^,^12^. To date, several TLRs including TLR3 and TLR4 have been identified in hMSCs^4^,^11^,^12^. TLR3 and TLR4 recognize polyinosinic-polycytidylic acid (poly(I:C)), a synthetic analog of double-stranded RNA that is structurally similar to double-stranded RNA in some viruses, and lipopolysaccharide (LPS), a major component of the cell walls of Gram-negative bacteria^13^,^14^,^15^,^16^. TLR-primed hMSCs release cytokines to interfere with dendritic cell and T-cell function, ultimately leading to protection of hMSCs against allorejection^3^,^17^,^18^. Therefore, the TLR-induced immunomodulatory response of hMSCs has received substantial attention in the field of hMSC-based therapies^3^,^17^,^18^,^19^,^20^. However, the mechanisms underlying TLR-induced cytokine release in hMSCs remain unknown and knowledge gaps exist between TLR-priming and cytokine release in hMSCs.

Extracellular Ca^2+^ entry through the plasma membrane and Ca^2+^ mobilization from intracellular stores leads to temporally and spatially distinct patterns of [Ca^2+^]_i_ to generate a highly versatile intracellular signal that controls almost all known molecular and cellular events in germ cells, somatic cells and stem cells^21^,^22^,^23^,^24^,^25^,^26^,^27^,^28^. These events include gene transcription, protein phosphorylation, exocytosis, endocytosis, migration, contraction, mitosis, proliferation, differentiation, survival, growth, inflammation, apoptosis and necrosis^21^,^22^,^23^,^24^,^25^,^26^,^27^,^28^. In general, various Ca^2+^ entry pathways, such as store-operated Ca^2+^ entry (SOCE), which is composed of Orai and STIM proteins or transient receptor potential (TRP) channels, as well as voltage-gated calcium channel (VGCC)-mediated Ca^2+^ influx operate in the plasma membrane^29^,^30^,^31^,^32^,^33^,^34^. Several intracellular organelles, e.g., the endoplasmic reticulum (ER) and nuclear envelope, store large amounts of Ca^2+^ and release these divalent ions through the ligand-gated calcium channels inositol triphosphate receptors (IP_3_Rs) and ryanodine receptors (RyRs)^27^,^35^,^36^,^37^. Different cell types can employ distinct sets of intracellular Ca^2+^-handling devices to control their [Ca^2+^]_i_ responses and the corresponding downstream molecular and cellular events^29^,^30^,^31^,^32^,^33^,^34^,^37^.

Ca^2+^ signaling is crucial for both proliferation and differentiation in several types of stem cells^38^,^39^,^40^,^41^. The ER Ca^2+^ stores function as a key intracellular Ca^2+^ source to generate the complex oscillation, fast transient increase and sustained elevation of [Ca^2+^]_i_ in various stem cells^39^,^42^,^43^,^44^,^45^. The ER Ca^2+^ channels IP_3_Rs and RyRs play an important role in the neuronal or cardiac differentiation of stem cells^39^,^43^,^44^,^45^. TRP channels mediate spontaneous Ca^2+^ transients to regulate both proliferation and differentiation in human neural progenitor cells^46^. Surprisingly, STIM1 participates in both early neural differentiation of embryonic stem cells and the survival of early differentiated embryonic stem cells independent of Orai1-mediated SOCE^38^. Indeed, a recent work revealed that Ca^2+^ mobilization from the ER and Ca^2+^ influx through TRPM7 channels occur in hMSCs to generate [Ca^2+^]_i_ oscillations upon application of mechanical traction on the plasma membrane^35^. It is well known that SOCE and Ca^2+^ mobilization from the ER regulate numerous activities, including TLR-mediated cytokine production in immune cells^31^,^47^,^48^. The aforementioned knowledge prompted us to determine whether [Ca^2+^]_i_ couples TLR-priming to cytokine release in hMSCs. The present work verifies that TLR3- and TLR4-priming differentially enhances Ca^2+^ signaling and cytokine expression and Ca^2+^ -dependently elevates cytokine release in hMSCs.

## Results

### Expression of stem cell markers and differentiation potential in hMSCs

**Figure 1:**
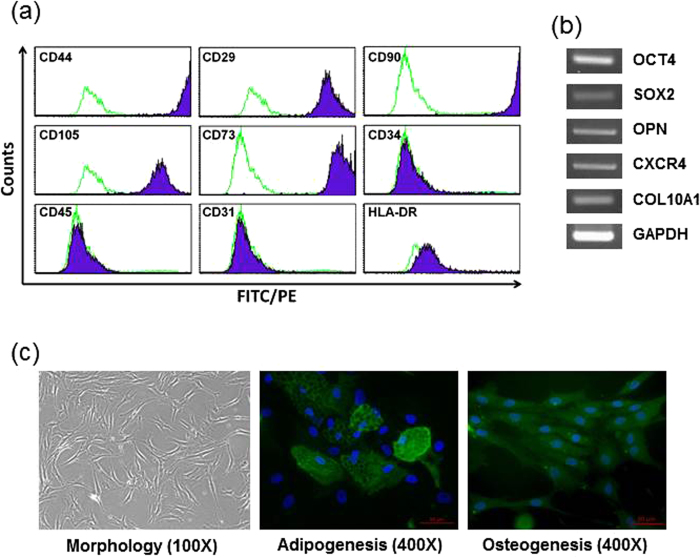
Characterization of TLR4-primed hMSCs. (**a**) Flow cytometry analysis represented the immunophenotype of hMSC. hMSCs expressed CD44, CD29, CD90, CD105 and CD73. (**b**) RT-PCR confirmation using stem cell marker genes. RT-PCR analysis used that stem cell markers OCT4, SOX2, OPN, CXCR4, and COL10A1. GAPDH was used as an endogenous control. (**c**) hMSC morphology in normal conditions (left) with 100X magnification. Differentiation potential into adipocytes (middle) or osteoblasts (right) was shown with 400X magnification. Adipocytes or osteoblasts were stained with FABP4 or osteocalcin antibody (green), and nuclei were counterstained with DAPI (blue).

MSC specific surface markers present on hMSCs were analyzed by flow cytometry (Fig. 1a). The cells were positive for an adhesion molecule (CD44), an integrin marker (CD29), and MSC markers (CD90, CD105, CD73), and were negative for a hematopoietic marker (CD34, CD45), an endothelial marker (CD31), and major histocompatibility antigen (HLA-DR). We performed RT-PCR analysis to quantitate the expression of stem cell specific genes. As shown in Fig. 1b, hMSCs expressed markers for pluripotency and self-renewal (OCT4, SOX2), osteogenic state (OPN), mesoderm state (CXCR4), and extracellular matrix molecules (COL10A1). The expression pattern of surface proteins and genes on our hMSC preparations indicated that these cells are primitive to the MSC population. To evaluate the differentiation ability of hMSCs, the cells were cultured in adipogenic and osteogenic medium for 3 weeks. Figure 1c shows the control hMSC morphology (left) and the capacity of hMSCs to differentiate into adipocytes (middle) and osteoblasts (right).

### Characterization of Basal [Ca^2+^]_i_ and Ca^2+^ Release and Entry Pathways in hMSCs

**Figure 2:**
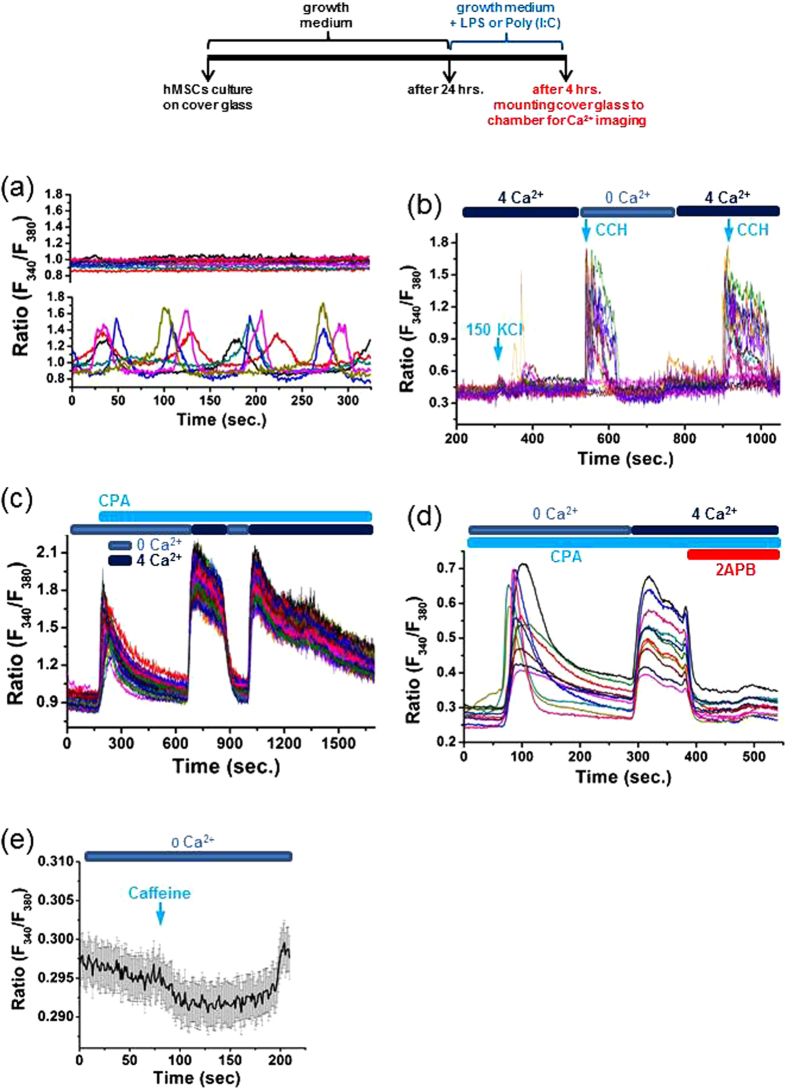
Basal [Ca^2+^]_i_ patterns and Ca^2+^ release and entry pathways in hMSCs. (**a**) hMSCs are classified into two populations, in terms of their basal [Ca^2+^]_i_ profiles, one showing spontaneous [Ca^2+^]_i_ oscillations (lower panel) and the other exhibiting a stable resting [Ca^2+^]_i_ (upper panel). (**b**) Representative [Ca^2+^]_i_ responses of hMSCs to 150 mM KCl and 50 μM carbachol (CCH) in the presence and absence of extracellular Ca^2+^. (**c**) Representative [Ca^2+^]_i_ alterations in hMSCs exposed to the SERCA inhibitor CPA (10 μM) with and without extracellular Ca^2+^. (**d**) Representative [Ca^2+^]_i_ traces showing [Ca^2+^]_i_ profiles following 10 μM CPA exposure in the absence of extracellular Ca^2+^. The Ca^2+^ influx evoked by extracellular addition of 4 mM Ca^2+^ and its abolishment by the membrane permeable SOCE antagonist 2APB (50 μM) in hMSCs. (**e**) [Ca^2+^]_i_ profiles in hMSCs treated with caffeine (10 mM) in the absence of extracellular Ca^2+^.

We first characterized basal [Ca^2+^]_i_ in hMSCs. Real-time single-cell measurements of [Ca^2+^]_i_ revealed two distinct patterns of basal [Ca^2+^]_i_ in hMSCs (Fig. 2a). Some hMSCs maintained a stable resting [Ca^2+^]_i_, whereas others displayed spontaneous [Ca^2+^]_i_ oscillations without stimulation.

We next clarified the mechanisms underlying Ca^2+^ mobilization from intracellular stores in hMSCs. Stimulation with the muscarinic agonist carbachol (CCH; 50 μM) drastically increased [Ca^2+^]_i_ in the absence and presence of extracellular Ca^2+^ (Fig. 2b). Importantly, the addition of 50 μM CCH evoked similar increases in [Ca^2+^]_i_ in the absence and presence of extracellular Ca^2+^. Such CCH-induced [Ca^2+^]_i_ increases suggest that Ca^2+^ mobilization from IP_3_-sensitive stores is operational in hMSCs. Furthermore, challenge with the RyR agonist caffeine (10 mM) did not cause a [Ca^2+^]_i_ increase, but instead slightly decreased [Ca^2+^]_i_ (Fig. 2e). The inability of caffeine to mobilize Ca^2+^ from intracellular stores verifies the presence of few, if any, caffeine/ryanodine-sensitive Ca^2+^ stores in hMSCs. These data reveal that hMSCs employ IP_3_-sensitive stores rather than caffeine/ryanodine-sensitive stores to release Ca^2+^ into the cytosolic compartment.

We also elucidated Ca^2+^ entry pathways in hMSCs. As shown in Fig. 2b, depolarization with 150 mM KCl produced no appreciable alteration in [Ca^2+^]_i_. This suggests that hMSCs do not use VGCC to mediate Ca^2+^ influx. Interestingly, extracellular Ca^2+^ efficiently entered hMSCs whose intracellular Ca^2+^ stores were depleted by inhibiting the sarcoendoplasmic reticulum Ca^2+^ ATPases with the SERCA inhibitor CPA (10 μM) (Fig. 2c,d). Moreover, such Ca^2+^ entry was effectively abolished by application of the membrane permeable SOCE antagonist 2APB (50 μM) (Fig. 2d). It is clear that extracellular Ca^2+^ enters hMSCs through SOCE. These observations illustrate that SOCE serves as an important mechanism to mediate Ca^2+^ entry through the plasma membrane of hMSCs.

### TLR3- and TLR4-Priming Up-Regulates the mRNA Expression Levels of TLR3, TLR4 and Cytokines in hMSCs

**Figure 3:**
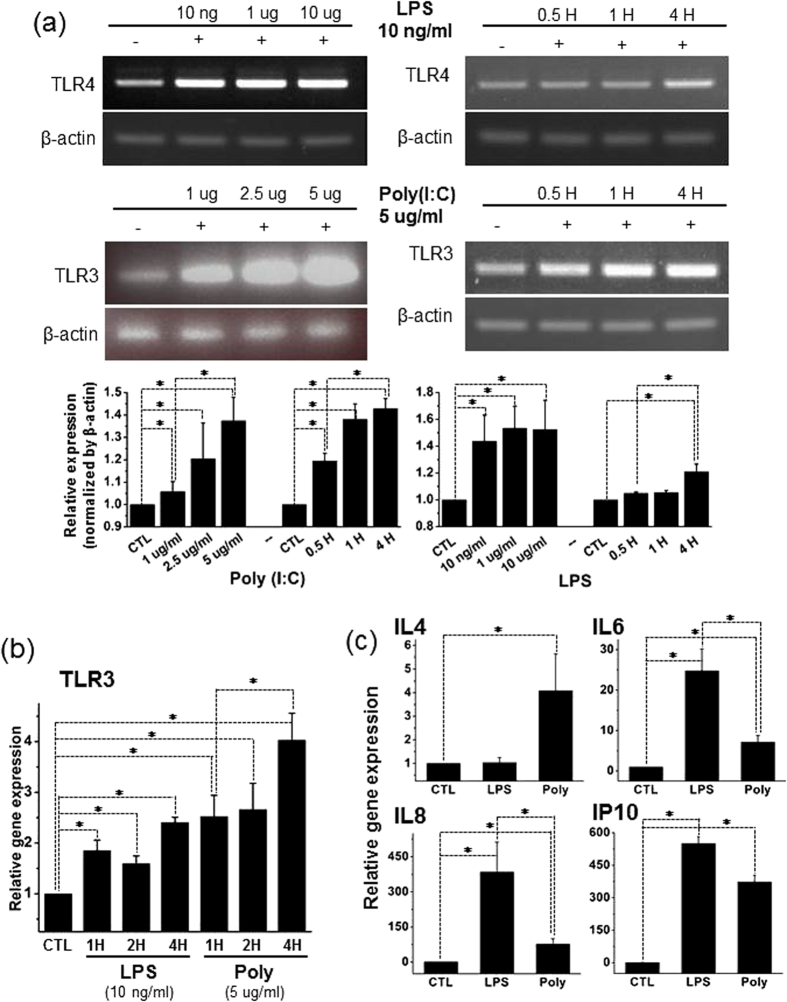
Exposure to LPS or Poly(I:C) elevates the mRNA expression of TLR3 and cytokines in hMSCs. (**a**,**b**) Conventional and real-time RT-PCR assays and quantification results showing that TLR3 and TLR4 mRNA expression levels were increased in a concentration and time dependent manner after exposure to LPS or poly(I:C). LPS (10 ng/ml) elevates TLR3 mRNA expression. Poly(I:C) concentration-dependently increases TLR3 mRNA expression. 4 h incubation with both ligands preferably elevated TLR3 and TLR4 mRNA levels. β-Actin serves as an internal control. (**c**) Real-time RT-PCR analysis revealing the 4 h incubation with LPS- and poly(I:C)-induced up-regulation of mRNA expression of cytokines including IL4, IL6, IL8 and IP10 in hMSCs. LPS preferably boosts IL6, IL8 and IP10 mRNA expression, whereas poly(I:C) raised only the mRNA level of IL4. The significance level was set at *p < 0.05.

To quantify the impact of the TLR3 agonist poly(I:C) and the TLR4 agonist LPS on the mRNA expression levels of TLR3, TLR4 and cytokines in hMSCs, we performed RT-PCR and real-time RT-PCR assays. RT-PCR analysis confirmed that control hMSCs expressed both TLR3 and TLR4 mRNAs. This analysis revealed that 4 h exposure to LPS and poly(I:C) elevated TLR4 and TLR3 mRNA expression in hMSCs in a concentration and time-dependent manner (Fig. 3a). Quantification data show the sum of triplicate repeated RT-PCR (Fig. 3a, lower panel). Neither poly(I:C) exposure nor LPS treatment influenced the expression of β-actin. Real-time RT-PCR showed that TLR3 mRNA levels reached the highest level in cells exposed to 5 μg/ml poly(I:C) for 4 h during different exposure times, whereas 1 h treatment with LPS (10 ng/ml) appeared to elevate TLR3 mRNA expression to a plateau level (Fig. 3b). These results suggest that TLR3 expression is more plastic than TLR4 expression following priming of the corresponding receptors.

Interestingly, real-time RT-PCR detection showed that incubation with 5 μg/ml poly(I:C) for 4 h preferably elevated IL4 mRNA levels. In contrast, 4 h treatment with LPS (10 ng/ml) preferentially up-regulated the mRNA expression levels of IL6, IL8 and IP10 (Fig. 3c). These findings reveal that TLR3- and TLR4-priming differentially regulate the mRNA expression of several cytokines including IL4, IL6, IL8 and IP10 in hMSCs.

### TLR3-Priming Potently Promotes IP_3_R Expression and IP_3_R-Mediated Ca^2+^ Mobilization in hMSCs

To explore the possible signaling pathways of TLR3 and TLR4 that respond to the highly versatile intracellular signal Ca^2+^, we focused our attention on Ca^2+^ mobilization from IP_3_-sensitive stores, which is likely to be the only Ca^2+^ release mechanism in hMSCs (Fig. 2). Therefore, we examined the effects of poly(I:C) and LPS treatments on ITPR (IP_3_R) expression and IP_3_R-mediated Ca^2+^ mobilization in hMSCs using RT-PCR analysis, [Ca^2+^]_i_ measurements, confocal immunofluorescence microscopy and western blot analysis.

**Figure 4:**
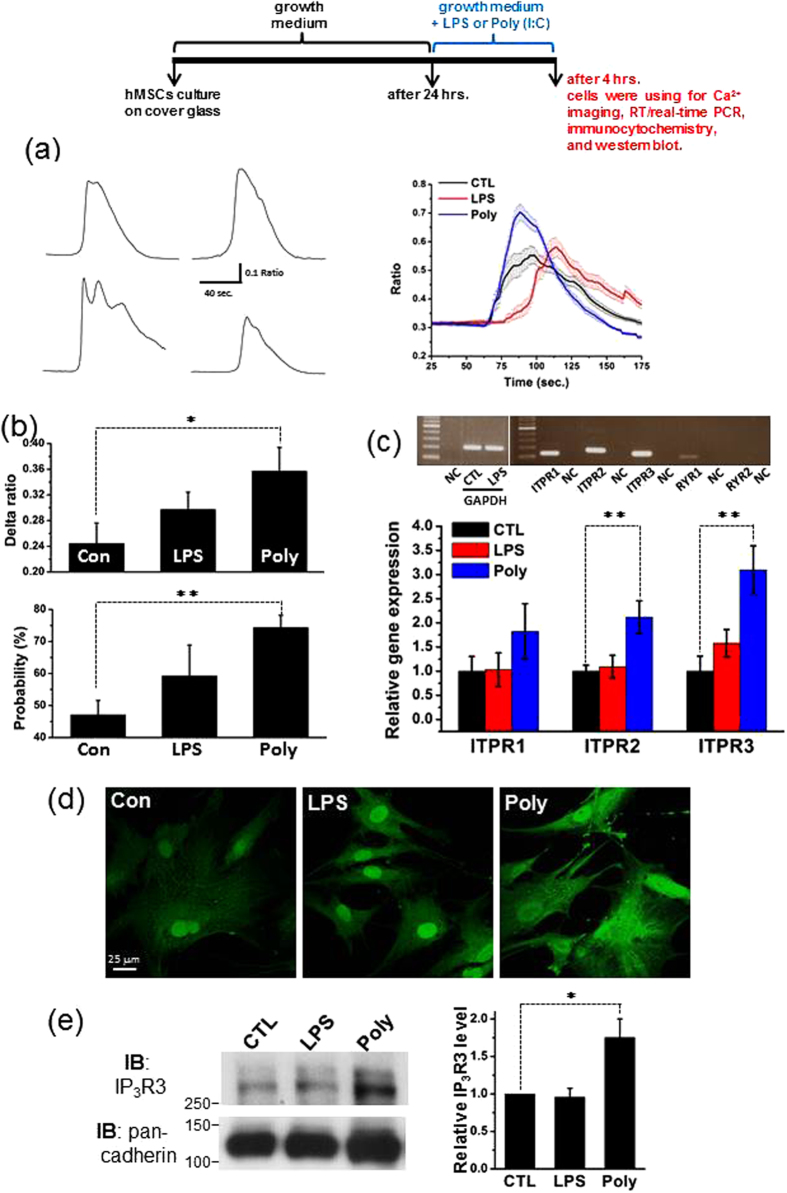
Treatment with LPS or Poly(I:C) increases IP_3_R expression and IP_3_R-mediated Ca^2+^ mobilization without influencing Dihydropyridine-Sensitive Ca^2+^ entry in hMSCs. (**a**) Representative [Ca^2+^]_i_ traces showing carbachol-evoked [Ca^2+^]_i_ transients registered in four individual cells bathed in extracellular solution without Ca^2+^ (left panel). Averaged [Ca^2+^]_i_ traces depicting the mean [Ca^2+^]_i_ responses to carbachol challenge in control cells (CTL; n = 26 cells) and cells treated with LPS (n = 43 cells) or poly(I:C) (n = 61 cells) in the absence of extracellular Ca^2+^. (**b**) Upper graph illustrating the mean net increases in [Ca^2+^]_i_ reflected by the averaged delta F340/F380 ratios obtained from control (CTL; n = 9), LPS- (n = 9) and poly(I:C)-treated groups (n = 9). Lower graph showing the averaged percentages of carbachol-responsive cells subjected to control treatment (n = 19), exposure to LPS (n = 19) and incubation with poly(I:C) (n = 19). Herein, n denotes the number of experiments. (**c**) Representative RT-PCR blots showing the mRNA expression of three IP_3_R subtypes (ITPR1, ITPR2 and ITPR3) and two RyR subtypes (RYR1 and RYR2) in control cells. GAPDH serves as an internal control. NC indicates negative control, i.e., distilled water. Real time RT-PCR quantification illustrating the different mRNA expression profiles of three IP_3_R subtypes (ITPR1, ITPR2 and ITPR3) in the control, LPS and poly(I:C) groups. Experiments were performed three times. (**d**) Confocal images showing the different intensities of IP_3_R3 immunofluorescence in control cells (left panel) and cells exposed to LPS (middle panel) or poly(I:C) (right panel). (**e**) Representative western blot of IP_3_R3 in control cells and cells exposed to LPS or poly(I:C) (left panel). Summarized graph showing the normalized level of IP_3_R in indicated conditions (right panel). Pan-Cadherin was used as a loading control. Experiments were performed six times. The significance level was set at *p < 0.05 or **p < 0.005.

[Ca^2+^]_i_ measurements showed that stimulation with 50 μM CCH evoked [Ca^2+^]_i_ transients with somewhat different patterns in control cells bathed in extracellular solution without Ca^2+^ (Fig. 4a, left panel). Incubation with 5 μg/ml poly(I:C) for 4 h significantly increased CCH-evoked [Ca^2+^]_i_ responses and the percentage of CCH-responsive cells in the absence of extracellular Ca^2+^ (Fig. 4a, right panel and Fig. 4b). However, treatment with 10 ng/ml LPS for 4 h only marginally elevated these two parameters under the same experimental conditions. These results illustrate that TLR3-priming potently promotes IP_3_R-mediated Ca^2+^ mobilization in hMSCs, but TLR4-priming is not potent enough to do so.

The RT-PCR blot shows that control hMSCs expressed abundant ITPR1 (IP_3_R1), ITPR2 (IP_3_R2) and ITPR3 (IP_3_R3) mRNAs, but very few RYR1 mRNAs and no RYR2 mRNAs (Fig. 4c, upper panel). The real-time RT-PCR assay showed that priming of TLR3 with poly(I:C) induced significant increases in ITPR1 (IP_3_R1), ITPR2 (IP_3_R2) and ITPR3 (IP_3_R3) mRNAs, whereas priming of TLR4 with LPS did not influence the mRNA expression of all three subtypes of IP_3_Rs (Fig. 4c, lower panel). Furthermore, confocal immunofluorescence microscopy revealed that TLR3-primed hMSCs (Fig. 4d, right panel) displayed more intense IP_3_R3 immunoreactivity than TLR4-primed (Fig. 4d, middle panel) and control hMSCs (Fig. 4d, left panel) under the same experimental conditions. There was no appreciable difference in the intensity of IP_3_R3 immunoreactivity between TLR4-primed and control hMSCs. Finally, western blot analysis was performed to examine the expression level of the IP_3_R in same condition. Consistent with the immunofluorescence results, treatment with poly(I:C), but not LPS, induced significant increases in the expression of the IP_3_R3 (Fig. 4e). These data verify that TLR3-priming rather than TLR4-priming is potent enough to enhance the expression of IP_3_Rs in hMSCs.

### TLR3-Priming Effectively Augments Orai and STIM Expression and SOCE in hMSCs

To reveal other possibilities that bridge TLR3- and TLR4 priming and [Ca^2+^]_i_, we also studied the hMSC-predominant Ca^2+^ influx SOCE. We evaluated the influence of TLR3- and TLR4-priming on the expression of three Orai and two STIM proteins as well as SOCE in hMSCs by RT-PCR analysis, [Ca^2+^]_i_ measurements, confocal immunofluorescence microscopy and western blot analysis.

**Figure 5:**
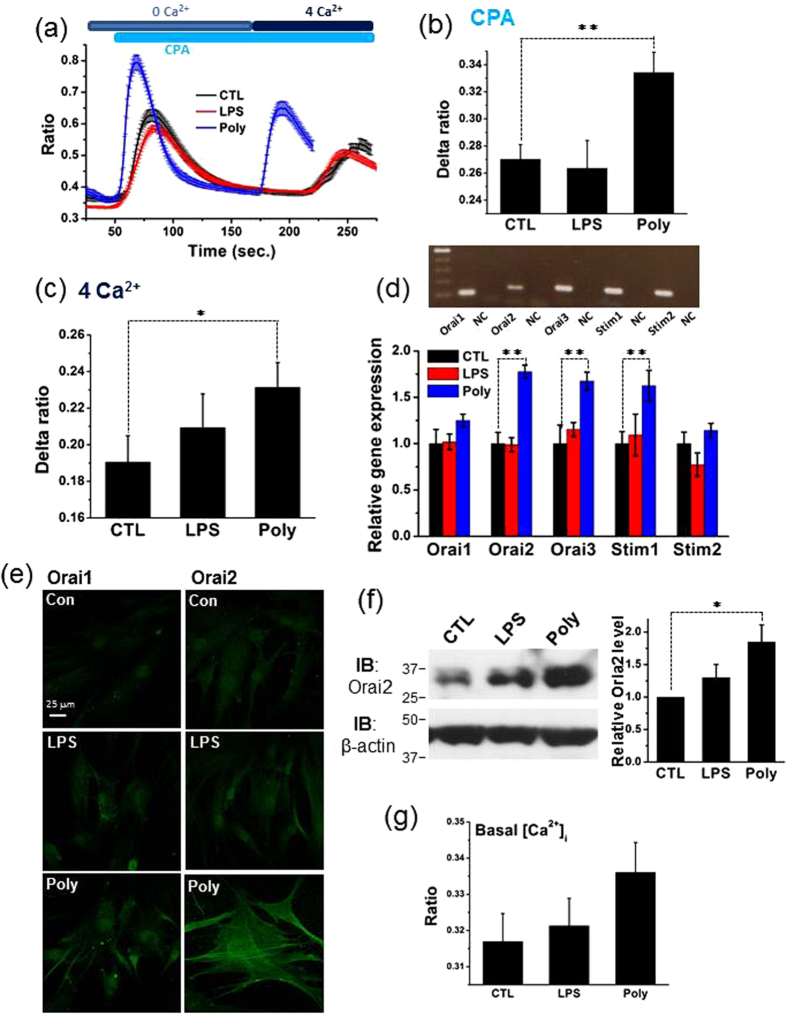
Incubation with Poly(I:C) rather than LPS augments Orai and STIM expression and SOCE in hMSCs. (**a**) Averaged [Ca^2+^]_i_ traces showing [Ca^2+^]_i_ transients induced by stimulation with CPA (first) and those evoked by addition of extracellular Ca^2+^ (second) in control (n = 40 cells), LPS- (n = 79 cells) and poly(I:C)-treated cells (n = 30 cells) immersed in Ca^2+^-free extracellular solution. (**b**) Summarized graph illustrating the mean net increases in [Ca^2+^]_i_ reflected by the averaged delta F340/F380 ratios recorded in control, LPS- or poly(I:C)-treated groups. Experiments were performed sixteen times. (**c**) Summarized graph showing the mean net increases in [Ca^2+^]_i_ reflected by the averaged delta F340/F380 ratios following extracellular application of 4 mM Ca^2+^ in control, LPS- or poly(I:C)-treated cells with intracellular Ca^2+^ stores pre-emptied by CPA. Experiments were performed sixteen times. (**d**) Representative RT-PCR blots (upper panel) illustrating the mRNA expression levels of three Orai subtypes and two STIM subtypes in control cells. NC represents the negative control with distilled water. Real-time RT-PCR quantification (lower panel) showing different mRNA expression profiles of three Orai subtypes (Orai1, Orai2 and Orai3) and two STIM subtypes (Stim1 and Stim2) in the control (n = 3), LPS (n = 3) and poly(I:C) (n = 3) groups. (**e**) Confocal images illustrating the different intensities of Orai1 and Orai2 immunofluorescence in control cells (upper panel) and cells exposed to LPS (middle panel) or poly(I:C) (lower panel). (**f**) Representative western blot of Orai2 in control cells and cells exposed to LPS or poly(I:C) (left panel). Summarized graph showing the normalized level of Orai2 in the indicated conditions. β-actin was used as a loading control. Experiments were performed four times (right panel). (**g**) Summarized graphs showing basal [Ca^2+^]_i_ reflected by the averaged F340/F380 ratios registered before application of CPA in control cells and cells exposed to LPS or poly(I:C). Experiments were performed nineteen times. The significance level was set at *p < 0.05 or **p < 0.005.

Single-cell [Ca^2+^]_i_ analysis revealed that exposure to CPA (10 μM) and the subsequent addition of extracellular Ca^2+^ evoked prominent [Ca^2+^]_i_ transients in control (n = 18), LPS- (n = 17) and poly(I:C)-treated groups (n = 17) in Ca^2+^-free extracellular solution (Fig. 5a). Importantly, the mean net increase of [Ca^2+^]_i_ reflected by the averaged delta F340/F380 ratios following CPA exposure was significantly higher in the poly(I:C) group than in the control group, whereas this parameter was similar between LPS-treated and control cells (Fig. 5b). More importantly, the mean net increase of [Ca^2+^]_i_ induced by extracellular application of 4 mM Ca^2+^ following Ca^2+^ store depletion by CPA was significantly exaggerated in poly(I:C)-treated cells, but just marginally elevated in LPS-treated cells in comparison with that in control cells (Fig. 5c). Moreover, basal [Ca^2+^]_i_ was mirrored by the averaged F340/F380 ratios prior to application of CPA and was increased significantly in the poly(I:C) group, but was elevated only slightly in LPS the group compared with the control group (Fig. 5g). There is no doubt that TLR3-priming effectively enhances SOCE with a concomitant increase in basal [Ca^2+^]_i_ in hMSCs.

The RT-PCR assay shows that the mRNA expression levels of three Orai subtypes and two STIM subtypes as well as TRPM4, TRPM7 and TRPC4 occurred clearly in control cells (Fig. 4d and Figure S1). Real-time RT-PCR analysis illustrates that the mRNA levels of two Orai subtypes and one STIM subtypes significantly elevated in the poly(I:C) group (n = 3), but not in the LPS group (n = 3) in comparison with the control group (n = 3) (Fig. 5d). In addition, the expression of the large-conductance calcium-activated potassium channel gene MaxiK did not change following treatment with either poly(I:C) or LPS in hMSCs (Figure S2). Furthermore, confocal immunofluorescence microscopy showed that Orai2 (ii) immunofluorescence was significantly brighter in TLR3-primed cells than in control cells under the same experimental conditions. In contrast, this immunofluorescence was only slightly brighter in TLR4-primed cells than in control cells (Fig. 5e). Because poly(I:C) treatment greatly enhanced the mRNA level of Orai2 among the members of SOCE, western blot analysis was employed to examine the protein level of Orai2 under the same condition. Consistent with the previous results, treatment with poly(I:C) but not LPS significantly increased the expression of Orai2 (Fig. 5f). Taken together, these findings suggest that TLR3-priming exaggerates SOCE-mediated Ca^2+^ signaling.

### TLR3- and TLR4-Priming that is Ca^2+^-Dependent Enhances Cytokine Release from hMSCs

Cytokine release is considered an important activity in TLR3- and TLR4-primed hMSCs. This led us to study whether the promotion of Ca^2+^ signaling by TLR3- and TLR4-priming influences cytokine release from hMSCs. We measured IL6, IL8, IP10 and RANTES from cells exposed to either LPS or poly(I:C) in comparison with control cells.

**Figure 6:**
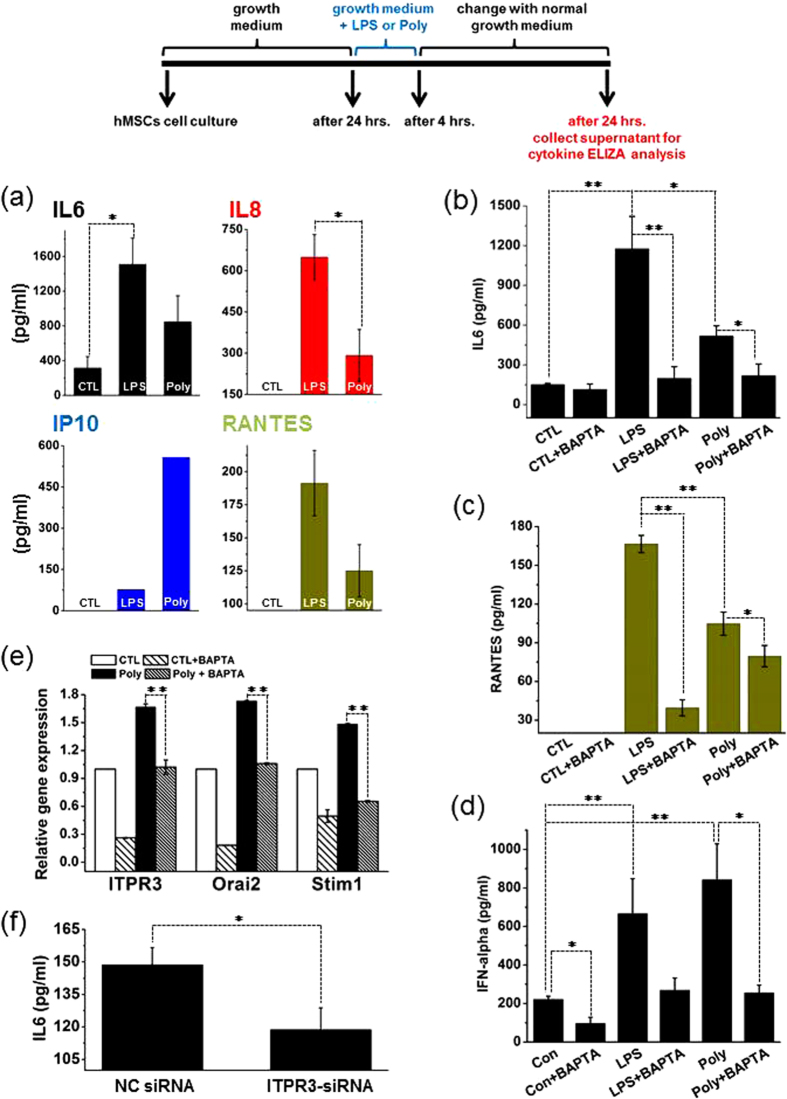
Stimulation with LPS or Poly(I:C) Promotes Cytokine Release in a Ca^2+^ Dependent Manner in hMSCs. (**a**) ELISA assay revealing more pronounced releases of IL6, IL8, IP10 and RANTES from cells exposed to LPS or poly(I:C) in comparison with control cells. Experiments were performed three times. (**b**–**d**) ELISA assay demonstrating the ablation of IL6, RANTES and IFN-alpha release by chelation of intracellular Ca^2+^ with BAPTA/AM (5 μM) and siRNA from LPS- or poly(I:C)-treated cells. Experiments were performed three times. (**e**) Real-time RT-PCR quantification showing ITPR3, Orai2 and Stim1 mRNA expression profiles in control and poly(I:C) with and without BAPTA/AM. Experiments were performed three times. (**f**) ELISA assay demonstrating the ablation of IL6 release by ITPR3 knockdown (ITPR3-siRNA). Experiments were performed six times. The significance level was set at *p < 0.05 or **p < 0.005.

ELISA assay shows that control cells released undetectable amounts of IL8, IP10 and RANTES, but measurable IL6 from control cells (Fig. 6a). Interestingly, TLR3- and TLR4-priming markedly promoted the release of IL6, IL8, IP10 and RANTES (Fig. 6a). More interestingly, TLR3- and TLR4-priming-induced release of IL6 and RANTES was effectively ablated by chelation of intracellular Ca^2+^ with BAPTA/AM (5 μM) (Fig. 6b,c). Type I interferons (IFNs) are mainly involved in the innate immune response against viral infection and have been identified as an important step in the initial inflammatory phase. We analyzed IFN-α and IFN-β cytokine release in TLR3- and TLR4-primed MSCs. Compared to untreated cells, IFN-α was increased in hMSCs following LPS and poly(I:C) treatment. Although the production of IFN-α by untreated cells was also increased. These unusually high constitutive productions of IFN-α are probably due in part to the differences in culture techniques. BAPTA/AM also showed a reducing effect on IFN-α release similar to IL6 and RANTES (Fig. 6d, upper panel). However, these factors did not induce the repression of IFN-β in hMSCs. We assessed the correlation between the BAPTA/AM effect and the mRNA expression of ITPR3, Orai2 and Stim1. Consistent with the cytokine results, real-time RT-PCR analysis illustrates that the mRNA levels of ITPR3, Orai2 and STIM1 were very significantly reduced by BAPTA/AM in both control and TLR3-primed hMSCs with similar patterns (Fig. 6e). These results show that enhanced cytokine release by TLR3- and TLR4-priming critically relies on [Ca^2+^]_i_ in hMSCs. We also investigated whether ITPR3 depletion (Figure S4) affects cytokine production. Using ELISA assay we found that compared to scrambled siRNA control (NC), IL6 in the supernatants was significantly decreased in ITPR3 siRNA hMSC cells (Fig. 6f).

## Discussion

The present work confirms that two different populations of hMSCs in the same extracellular milieu show two distinct profiles of basal [Ca^2+^]_i_, one exhibiting a stable resting [Ca^2+^]_i_ and the other displaying spontaneous [Ca^2+^]_i_ oscillations. It is plausible to postulate that one or more [Ca^2+^]_i_ oscillation-generating devices, e.g., IP_3_R2, somehow become active under basal conditions to create this spontaneous [Ca^2+^]_i_ oscillation in the latter population of hMSCs. This postulation is supported by the fact that the IP_3_R2 undergoes activation at the lowest concentration of IP_3_ among the three IP_3_R subtypes to generate [Ca^2+^]_i_ oscillations^48^ and that all three IP_3_R subtypes are present in hMSCs (see below for details). The distinction in the basal [Ca^2+^]_i_ profile may serve as a clue for the important aspects, such as cell cycle phase, differentiation fate, self-renewal capacity or immune-modulating features, of hMSCs. These two basal [Ca^2+^]_i_ profiles deserve further investigation to clarify what exactly they signify.

The results presented herein demonstrate that hMSCs prefer to use IP_3_-sensitive stores rather than engage caffeine/ryanodine-sensitive stores to mobilize stored Ca^2+^ into the cytosolic compartment. Indeed, our data show that hMSCs rely on IP_3_Rs rather than RyRs to mediate intracellular Ca^2+^ mobilization and are consistently supported by the molecular evidence that hMSCs abundantly express three subtypes of IP_3_Rs with sparse RyR1 and no RyR2 as shown in the present work and previously^42^. IP_3_Rs have been demonstrated to play important roles in embryonic development^49^,^50^. For example, IP_3_R1 is critical for neuronal development and IP_3_R3 is responsible for a proper pattern of [Ca^2+^]_i_ oscillations to negatively regulates apoptosis in early differentiating embryonic stem cells^49^,^50^. These previous findings prompt us to extrapolate that IP_3_Rs are important signaling devices in hMSC development, although their exact roles remain to be explored. In addition, the present work also corroborates that hMSCs acquire Ca^2+^ from the extracellular environment through SOCE rather than VGCCs. These functional observations are in accordance with previous studies^42^,^51^. However, the molecular identity of SOCE in hMSCs has not been convincingly determined, although TRPC4 was detected^42^. Strikingly, the present work not only showed TRPM4 and TRPM7 in addition to TRPC4 but for the first time also visualizes three subtypes of Orais and two subtypes of STIMs in hMSCs. This adds new molecular building blocks for the assembly of SOCE in hMSCs.

hMSCs appropriately sense and efficiently respond to environmental challenges and adequately engraft in inflamed sites or lesion areas to accomplish repair missions^3^,^13^,^14^,^15^,^16^,^17^,^18^,^52^. In fact, this is an immune modulating event where TLRs act as key players by recognizing natural ligands, such as the double-stranded RNA of viruses and bacterial cell-surface LPS, and trigger corresponding downstream signaling, e.g., cytokine release^3^,^13^,^14^,^15^,^16^,^17^,^18^. The present work focused on the most important two TLRs, TLR3 and TLR4^4^,^10^,^11^,^12^. Their priming can polarize hMSCs into two distinct populations, TLR4-primed hMSCs (MSC1) and TLR3-primed hMSCs (MSC2)^11^. Importantly, we revealed that the TLR3 agonist poly(I:C) boosts TLR3 mRNA expression in a concentration dependent manner, whereas the TLR4 agonist LPS just produces a marginal elevation in TLR4 mRNA but an appreciable increase in TLR3 mRNA in hMSCs. It is well known that TLR3 and TLR4 undergo allosteric alteration, dimerization and genomic up-regulation upon their activation in other cell types^14^,^15^. Our findings suggest that poly(I:C) and LPS activate TLR3 and TLR4 via different mechanisms in hMSCs; TLR3 activation involves a genomic mechanism in addition to allosteric alteration and dimerization, whereas TLR4 activation relies on only allosteric alteration and dimerization. It is noteworthy that the TLR4 agonist LPS markedly increases TLR3 expression without altering TLR4 expression. This means that LPS transactivates TLR3 because TLR3- and TLR4-primed hMSCs differ in various aspects, including the mRNA expression of IL4, IL6, IL8 and IP10 as revealed in the present work. This strongly supports that TLR3- and TLR4-primed hMSCs execute different immune modulating functions.

The present work has dissected the mechanisms linking TLR3 and TLR4 to [Ca^2+^]_i_. More importantly, we reveal that TLR3-priming produces not only a significant increase in IP_3_R-mediated Ca^2+^ mobilization but also a substantial elevation of the molecular expression of IP_3_Rs in hMSCs. In contrast, TLR4-priming has only marginal influences on these two parameters. Likewise, TLR3-priming significantly augments SOCE with a concomitant increase in basal [Ca^2+^]_i_ and the molecular expression of candidate building blocks of SOCE, including two Orai subtypes and one STIM subtypes as well as TRPM4 and TRPC4 in hMSCs. However, TLR4-priming fails to do so. These findings demonstrate that TLR3-priming but not TLR4-priming exaggerates IP_3_R- and SOCE-mediated Ca^2+^ signaling. They also suggest that TLR3-priming does not allosterically modulate IP_3_R and SOCE activity, but instead increases their abundance via genomic mechanisms. In addition to these Ca^2+^ channels, K^+^ channels are also present in hMSCs^53^,^54^. The channel-mediated K^+^ efflux causes a more negative membrane potential and thereby enhances Ca^2+^ influx due to the increased electric driving force for Ca^2+^ entry^37^. It is possible that TLR3-priming may up-regulate [Ca^2+^]_i_ through the increased expression of these K^+^ channels. Therefore, we have quantified the mRNA expression of the large-conductance calcium-activated potassium channel gene MaxiK^55^. Neither TLR3- nor TLR-4-priming influences MaxiK expression. Even so, this is particularly interesting because these negative data confirm the relatively selective regulation of TLR-3-priming on IP_3_Rs and SOCE.

Using RNA-sequencing analysis, we observed that 21 Ca^2+^ related signaling genes were significantly up-regulated in response to poly(I:C) and strongly correlated with calcium ion transport (Figure S3). In addition, we found that the putative binding sites for four transcription factors (TFs) were significantly enriched suggesting that these TFs might be involved in the regulation of Ca^2+^ signaling genes in TLR3 primed hMSCs. However, we could not observe a significant up-regulation of ITPR3 and STIM1 genes in our RNA-sequencing analysis. Further experiments are required to identify these genes during TLR3 primed hMSCs.

Most importantly, the present work demonstrates that TLR3- and TLR4-priming markedly and differentially enhances cytokine releases in a Ca^2+^-dependent fashion in hMSCs. It seems paradoxical that TLR4-priming elevates neither [Ca^2+^]_i_ nor the molecular expression of IP_3_Rs and SOCE but significantly increases cytokine release, which is diminished by chelation of intracellular Ca^2+^. In fact, this can be explained by the possibility that TLR4-priming acts at other steps in the complex process of cytokine release rather than [Ca^2+^]_i_ or the molecular expression of IP_3_Rs and SOCE^13^,^14^,^15^,^16^,^17^,^18^. Interestingly, in our study, we observed that BAPTA/AM have a much stronger effect on TLR4-primed IL6 and RANTES production than on the TLR3-primed cytokine production. TLR3 primarily activates the TIR-domain-containing adaptor inducing interferon β (TRIF)-dependent pathway, whereas TLR4 activates both myeloid differentiation factor 88 (MyD88) and TRIF dependent pathways^56^. Several studies have reported that the TLR4-MyD88 pathway has been thought to have an important role in TLR4-primed IL6 synthesis^57^,^58^. It is therefore likely that BAPTA/AM-modulated IL6 and RANTES production depends on both MyD88 and TRIF-dependent pathways rather than only the TRIF-dependent pathway. A better understanding of the consequences of TLR3 or TLR4-primed cytokines/chemokines production modulated by BAPTA/AM in hMSCs warrants a comprehensive investigation.

In conclusion, we verified that hMSCs mainly engage Ca^2+^ mobilization from IP_3_-sensitive stores and extracellular Ca^2+^ entry through SOCE to evoke [Ca^2+^]_i_ responses. These two Ca^2+^ -handling mechanisms undergo differential increases concomitant with the elevation of cytokine production upon TLR3- and TLR4-priming. TLR3- and TLR4-priming-induced cytokine release critically depends on [Ca^2+^]_i_. These findings not only clarify the novel signaling cascade from TLR3- and TLR4-priming via [Ca^2+^]_i_ to cytokine release, but also implicate potential targets for genetic and pharmacological manipulation in hMSC-based therapy.

## Methods

### hMSC Culture and Treatments

Experiments were performed using human bone marrow MSC which were derived from one donor, a black 22 year old female, these cells were purchased from Lonza (donor 7F3674; Walkersville, MD). hMSCs cultured in low-glucose Dulbecco’s modified eagle’s medium (DMEM; Gibco, Carlsbad, CA) supplemented with 10% fetal bovine serum (FBS; Hyclone, Logan, UT) and 100 U/100 μg/ml penicillin/streptomycin (Gibco, Carlsbad, CA) at 37 °C in a humidified 5% CO_2_ incubator. The cells were fed with fresh medium every 3–4 days and used at passages 5 and 6. hMSCs were incubated with LPS (10 ng/ml, TLR4-primed, Sigma Aldrich, St. Louis, MO) and poly(I:C) (1, 2 and 5 μM/ml, TLR3-primed, Sigma Aldrich, St. Louis, MO) in the culture medium for 4 h.

### RT-PCR Assays

Total RNA was extracted from hMSCs using RNAiso Plus (Takara, Shiga, Japan) according to the manufacturer’s instructions. The obtained RNA was reverse-transcribed with PrimeScript Reverse Transcriptase (Takara, Shiga, Japan). Subsequently, the resultant cDNA was amplified using SYBR Premix Ex Taq^TM^ II (Takara, Shiga, Japan). RT-PCR primer pairs were synthesized by GenoTech (Daejeon, Korea) and their sequences were listed in Table 1. Quantitative real-time PCR was performed on an ABI 7500 real-time PCR system (Applied Biosystems Inc., Carlsbad, CA) using the following parameters: initial denature at 95 °C for 10 min, followed by 40 cycles of 15 s at 95 °C and 1 min at 60 °C. Glyceraldehyde-3-phosphate dehydrogenase (GAPDH) was used as an internal control for quantitative analysis. The data were analyzed using the critical threshold (ΔCT) and the comparative critical threshold (ΔΔCT) methods in the AB-7500 software. Conventional PCR was carried out with S1000^TM^ Thermal Cycler (Bio-Rad, Hercules, CA) under the following conditions: initial denature at 95 °C for 5 min, followed by 30–35 cycles of denaturing at 95 °C for 1 min, annealing at 60 °C for 1 min and extending at 72 °C for 1 min. The amplified PCR products were detected by agarose gel electrophoresis and ethidium bromide staining.

### [Ca^2+^]_i_ Measurement

hMSCs attached to glass coverslips were incubated with TLR ligands for 4 h and then loaded with 2 μM fura-2/AM for 30 min at 37 °C in a physiological external solution consisting of (in mM) 138 NaCl, 5.6 KCl, 1 MgCl_2_, 10 HEPES and 10 glucose (pH 7.4). After loading, cells on the coverslips were transferred to an open perfusion chamber maintained at 37 °C. [Ca^2+^]_i_ was measured as the fura-2 340/380 nm fluorescence ratio with a fluorescence microscope (Nikon, Tokyo, Japan). The microscope was equipped with a xenon arc lamp, integrated shutter and cooled EM-CCD camera (ImagEM X2, Hamamatsu, Japan). The camera and shutter were controlled by MetaFluor software (Molecular Devices, Foster City, CA). Single cells were defined as regions of interest (ROIs) (Fig. 1A). The 16-bit grayscale images with a binning of 1 × 1 were captured every 1 s with an exposure time ranging from 100 to 300 ms. ROI signals were calculated by subtracting the background noise signals and the analyzed with MetaFluor software.

### Flow cytometry analysis of hMSCs

To stain the hMSCs, the cells were harvested with trypsin, and then blocked with phosphate buffered saline (PBS) with 2% normal serum for 5 min. The cells were incubated with direct immunofluorescence using fluorescein isothiocyanate (FITC)-conjugated antibodies against CD105 (Serotec Ltd., Oxford, U.K), HLA-DR (Serotec Ltd., Oxford, U.K), CD29 (Serotec Ltd., Oxford, U.K), CD44 (Dakocytomation, Glostrup, Denmark), and phycoerythrin (PE)-conjugated antibodies against CD34 (Serotec Ltd., Oxford, U.K), CD45 (DakoCytomation, Glostrup, Denmark), CD31 (DakoCytomation, Glostrup, Denmark), CD73 (BD Pharmingen, San Diego, CA), and CD90 (BD Pharmingen, San Diego, CA) for 30 min. Control cells were prepared with FITC-, PE-mouse isotype antibodies (Serotec Ltd., Oxford, U.K). The stained cells were analyzed with a FACS Calibur A (BD Bioscience, San Diego, CA).

### Differentiation of hMSCs into adipocytes and osteoblasts

The human mesenchymal stem cell functional identification kit (R&D systems, Minneapolis, MN) was employed for the differentiation of hMSCs into adipocytes and osteoblasts. Briefly, hMSCs were cultured in minimum essential medium (MEM, Gibco, Carlsbad, CA) containing adipogenic and osteogenic supplements for 21 days to induce differentiation into adipocytes and osteoblasts respectively. The medium was replaced with fresh medium every 3–4 days. After 21 days, differentiated cells were fixed with 4% paraformaldehyde and incubated with a primary antibody against fatty acid binding protein 4 (FABP4, 10 ug/ml, R&D Systems, Minneapolis, MN) for adipocytes and osteocalcin (10 ug/ml, R&D Systems, Minneapolis, MN) for osteoblasts. The cells were washed and incubated with fluorescein-labeled anti-rabbit IgG (Jackson ImmunoResearch, West Grove, PA). Stained cells were observed using a microscope (Nikon, Tokyo, Japan).

### Immunocytochemistry and Confocal Microscopy

hMSCs were seeded onto coverslips in 4-well plates, cultured for a day and treated with LPS or poly(I:C). Subsequently, the cells were washed with phosphate-buffered saline (PBS), fixed with 4% paraformaldehyde in PBS for 15 min and permeabilized with cold methanol for 5 min. Then, the samples were blocked with 3% bovine serum albumin for 1 h, incubated with rabbit polyclonal anti-IP_3_R3 (1:100; Abcam, Cambridge, UK), rabbit polyclonal anti-Orai1 (1:100; Abcam) or rabbit polyclonal anti-Orai2 (1:100; Abcam) at 4 °C overnight. A subsequent incubation of the samples with Goat anti-rabbit IgG conjugated to Alexa 488 (1:100; Life Technologies, Carlsbad, CA) was performed for 30 min at 37 °C. Finally, the samples were mounted in the mounting medium Vectashield (Vector Laboratories, Burlingame, CA) and visualized with a Zeiss LSM 710 confocal microscope (Jena, Germany).

### Western blot analysis

Cell lysates were prepared by sonication in lysis buffer containing 50 mM Tris-HCl, pH 7.4, 150 mM NaCl, 2 mM EDTA, 5 mM MgCl_2_, 1% Triton X-100 and a complete protease inhibitor mixture tablet (Roche Applied Science). Equal amounts of protein (30–50 μg) were subjected to 6 or 10% SDS-PAGE and blotted onto a PVDF membrane. The membranes were then incubated with anti-IP_3_R3 (1:1000; Abcam) or anti-Orai2 (1:1000; Abcam) and anti-pancadherin (1:1000; Abcam) or anti-β-actin (1:10000; Sigma) for loading controls and signals were detected with ECL reagent.

### ELISA Detection of Cytokines

hMSCs were seeded at a density of 6 × 10^4^ cells/cm^2^ into 10 cm dish containing culture medium and cultivated for a day. Thereafter, the normal medium was replaced with the medium supplemented with TLR ligands followed by a 4 h incubation. Cytokine concentrations in the culture media were detected by enzyme-linked immunosorbent assay (ELISA) according to the manufacturer’s instructions. Standard curves were established using mouse recombinant cytokines provided with the ELISA kit (KOMABIOTECH, Seoul, Korea and Elabscience Biotechnology, Beijing, China). The assay detection limit was 16 to 32 pg/ml.

### Statistical Analysis

Data are reported as mean ± SEM. Statistical analyses were performed using the Origin program (SPSS Inc., Chicago, IL). Statistical significance was determined by Student’s t test. The significance level was set at *p < 0.05 or **p < 0.005.

## Additional Information

**How to cite this article**: Park, K. S. *et al.* TLR3-/4-Priming Differentially Promotes Ca^2+^ Signaling and Cytokine Expression and Ca^2+^-Dependently Augments Cytokine Release in hMSCs. *Sci. Rep.* **6**, 23103; doi: 10.1038/srep23103 (2016).

## Supplementary Material

Supplementary Information

## Figures and Tables

**Table 1:** List of primers used for PCR.

Gene	Acc. No.	Forward primer (5′-3′)	Reverse primer (5′-3′)	bp
*TLR3*	NM003265	TTGCCTTGTATCTACTTTTGGGG	TCAACACTGTTATGTTTGTGGGT	157
*TLR4*	NM138557	AGACTGTCCCTGAACCCTAT	CGATGGACTTCTAAACCAGCCA	147
*ITPR1*	NM001168272	ATTGCTGGGGACCGTAATCC	TCCAATGTGACTCTCATGGCA	129
*ITPR2*	NM002223	CACCTTGGGGTTAGTGGATGA	CTCGGTGTGGTTCCCTTGT	172
*ITPR3*	NM002224	CCAAGCAGACTAAGCAGGACA	ACACTGCCATACTTCACGACA	142
*RyR1*	NM000531	CACCAATGGCCTATACAACCAG	GCTCAGGATAACGCCCTCG	115
*RyR2*	NM001035	GGCAGCCCAAGGGTATCTC	ACACAGCGCCACCTTCATAAT	137
*Orai1*	NM032790	GACTGGATCGGCCAGAGTTAC	GTCCGGCTGGAGGCTTTAAG	116
*Orai2*	NM001126340	GAGGCCGTGAGCAACATCC	GGAGGAACTTGATCCAGCAGA	157
*Orai3*	NM152288	GTGTCTGCTGCCCCACATT	GGCACAAACTTGACCCAACC	177
*Stim1*	NM003156	TGTGGAGCTGCCTCAGTATG	AAGAGAGGAGGCCCAAAGAG	183
*Stim2*	NM001169117	CACGCCCACCTCATAACTGG	TCAAGCCTCTCTTGTAAGTCCA	187
*TRPM4*	NM017636	GCACGACGTTCATAGTTGACT	CTTCTCCGTGGTGTGTGCAT	152
*TRPM7*	NM017672	ACTGGAGGAGTAAACACAGGT	TGGAGCTATTCCGATAGTGCAA	102
*TRPC4*	NM015638	CTCTATCCGGGGACAGAAACT	CAAACGCTTTGTAACTCCCTCT	106
*TRPC5*	NM012471	CTCTCGCTCCCGACTGAAC	GAAGGCAGTTAGGATGGGGTC	88
*GAPDH*	NM001256799	AAGGTCGGAGTCAACGGATT	CTCCTGGAAGATGGTGATGG	225
