# Supplementary material for: TLR3-/4-Priming Differentially Promotes Ca2+ Signaling and Cytokine Expression and Ca2+-Dependently Augments Cytokine Release in hMSCs
Source: Sci Rep. 2016 Mar 16;6:23103. doi: 10.1038/srep23103 (PMC4793222; doi:10.1038/srep23103)
Supplement: Supplementary Information [file srep23103-s1.pdf]

Supplemental Figures

TLR3-/4-Priming Differentially Promote Ca^{2+} Signaling and Cytokine Expression and Ca^{2+}-Dependently Augment Cytokine Release in hMSCs

Kyoung Sun Park[1], Sun Hwa Kim[1], Amitabh Das[1], Shao-Nian Yang[3], Kyoung Hwa Jung[1], Mi Kyung Kim[2], Per-Olof Berggren[3], YoungSeek Lee[1], Jin Choul Chai[1]Hyun Jin Kim*[2], YoungGyu Chai*[1]

[1]Department of Molecular and Life Sciences, Hanyang University, Ansan, Korea
[2]Department of Physiology, Sungkyunkwan University School of Medicine, Suwon, Korea
[3]The Rolf Luft Research Center for Diabetes and Endocrinology, Karolinska Institutet, Karolinska University Hospital L1, SE-171 76 Stockholm, Sweden

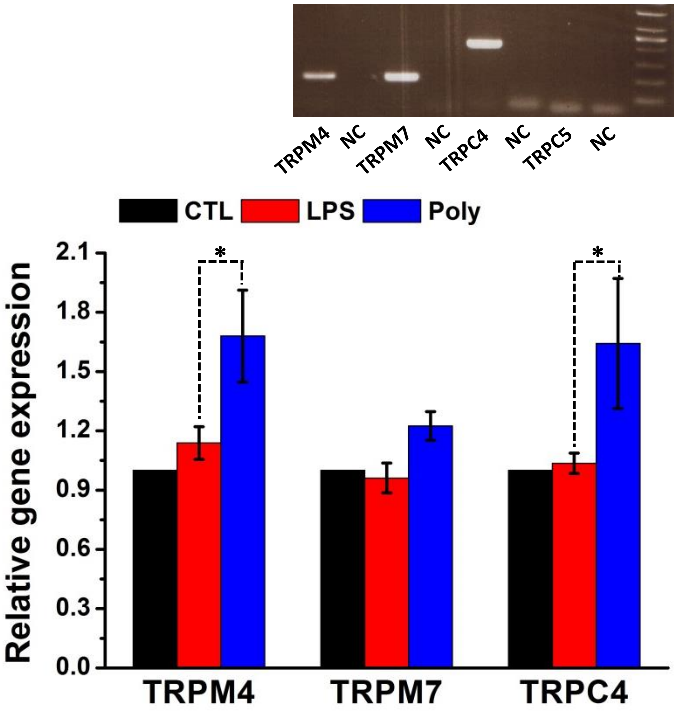

Figure S1. Representative RT-PCR blot illustrating mRNA expression of TRPM4, TRPM7 and TRPC4 in control hMSCs. NC denotes negative control with distilled water. Real time RT-PCR quantification showing relative mRNA expression levels of TRPM4, TRPM7 and TRPC4 in control (CTL; n = 5), LPS- (n = 5) and poly (I:C)-treated hMSCs (n = 5).

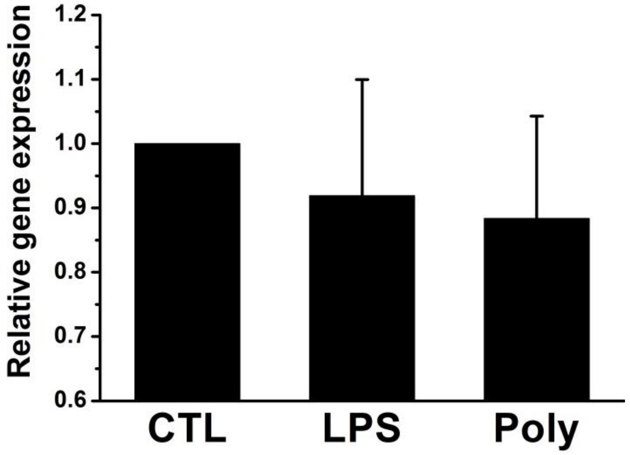

Figure S2. Real time RT-PCR quantification showing relative mRNA expression levels of MaxiK in control (CTL; n = 3), LPS- (n = 3) and poly (I:C)-treated hMSCs (n = 3).

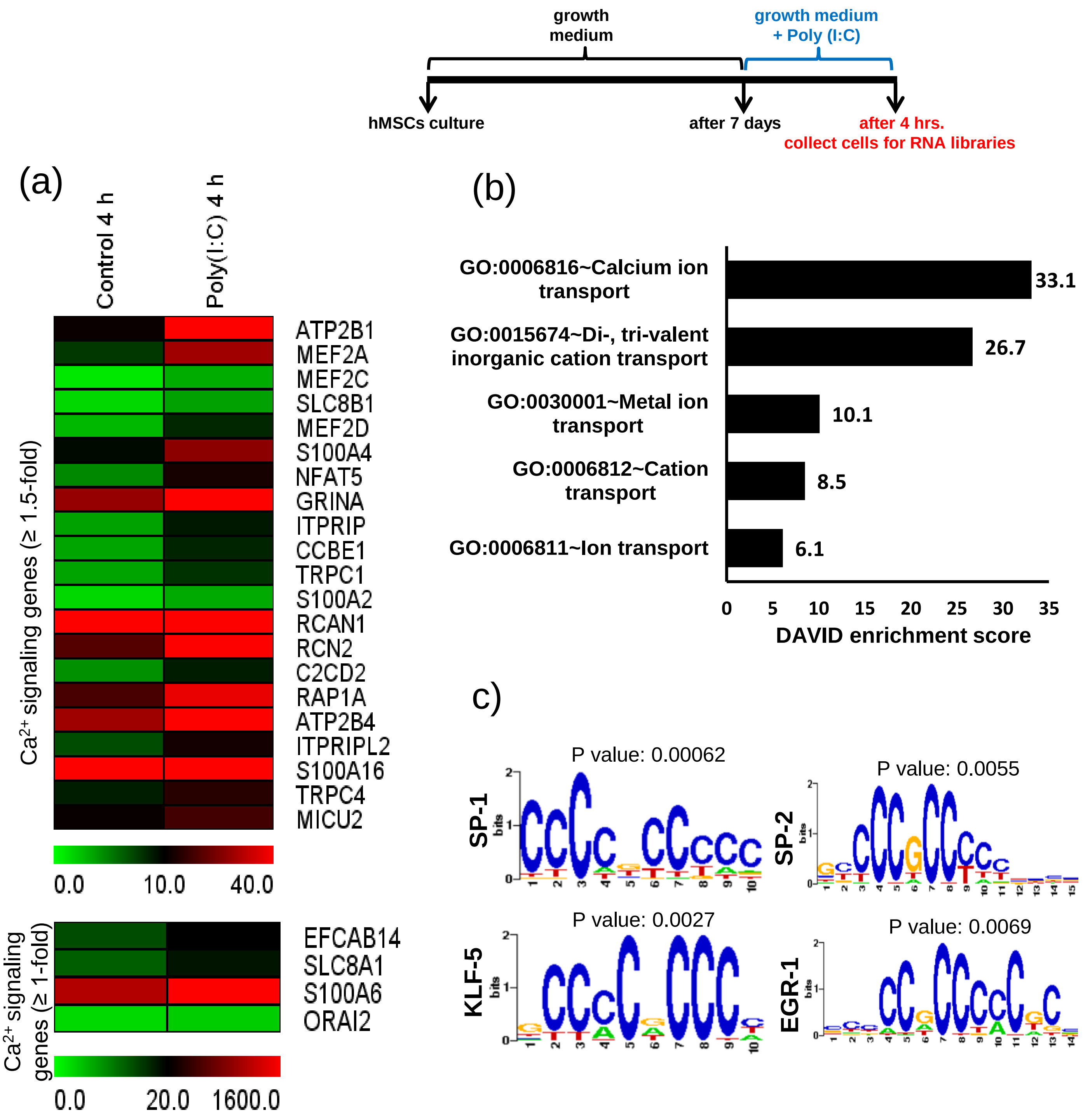

Figure S3. Transcriptomic analysis of the Poly (I:C)-stimulated Ca^{2+} signaling gene expression in hMSCs

(a) A heat map represents differential expression of Ca^{2+} signaling gene expression at 4 h after Poly (I:C) stimulation in hMSCs. (b) Gene ontology analysis of the functional annotations that were associated with Ca^{2+} signaling gene expression at 4 h after Poly (I:C) stimulation in hMSCs. (c) Patterns of transcription factor motif enrichments within the promoters of the Ca^{2+} signaling genes in Poly (I:C)-stimulated hMSCs.

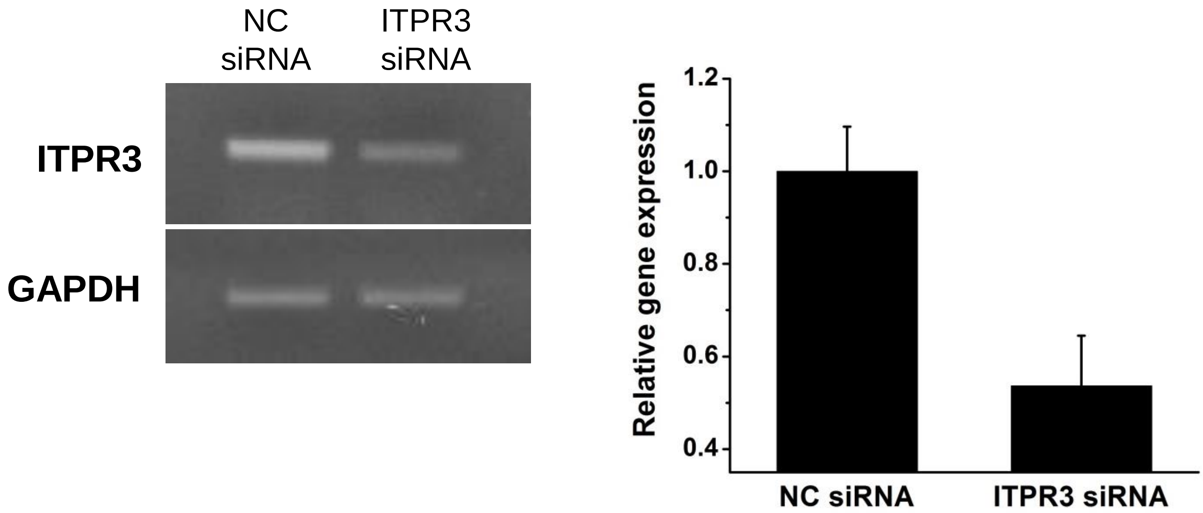

Figure S4. Representative RT-PCR blot and real time RT-PCR quantification showing relative mRNA expression levels of ITPR3 in scambled siRNA control (NC, n = 2) and ITPR3 siRNA treated hMSCs for 48 hours (n = 2).
